# Sequence-dependent real-world efficacy of anti-TNF antibodies and vedolizumab in ulcerative colitis: a propensity score-weighted cohort study

**DOI:** 10.1186/s12876-026-05560-4

**Published:** 2026-11-14

**Authors:** Fabienne Assel, Sebastian Schramm, Tanja M. Müller, Sophie Haberkamp, Rachel Rouse-Merkel, Raja Atreya, Markus F. Neurath, Sebastian Zundler

**Affiliations:** 1 Department of Medicine 1, University Hospital Erlangen, Friedrich-Alexander-Universität Erlangen-Nürnberg, Erlangen, Germany; 2 Deutsches Zentrum Immuntherapie (DZI), University Hospital Erlangen, Erlangen, Germany

**Keywords:** Ulcerative colitis, Sequential therapy, Vedolizumab, Anti-TNF

## Abstract

**Background:**

Previous exposure to advanced treatments for IBD is considered to reduce remission rates in further therapy lines and optimal treatment sequences remain to be established.

**Methods:**

We performed a retrospective propensity score-weighted cohort study including a total of 219 patients (281 therapy lines) with ulcerative colitis (UC) to compare the efficacy of first-line vedolizumab with second-line vedolizumab after previous anti-TNF and vice versa.

**Results:**

While second-line vedolizumab after first-line anti-TNF resulted in clearly lower rates of clinical and steroid-free remission after 52 weeks compared with first-line vedolizumab, no statistically significant reduction in clinical or steroid-free remission was detected for second-line anti-TNF after first-line vedolizumab compared with first-line anti-TNF.

**Conclusion:**

Exposure to vedolizumab was not associated with a statistically significant reduction in the effectiveness of subsequent anti-TNF antibodies in second-line therapy of UC, whereas prior exposure to anti-TNF antibodies was associated with a statistically significant reduction in the effectiveness of subsequent vedolizumab. These findings support considering vedolizumab as a first-line treatment option, although direct comparisons of treatment sequences are needed to determine the optimal treatment strategy.

**Supplementary information:**

The online version contains supplementary material available at https://doi.org/10.1186/s12876-026-05560-4.

## Background

Despite an increasing armamentarium of therapeutic options available for the medical treatment of inflammatory bowel diseases (IBD) such as Crohn’s disease (CD) or ulcerative colitis (UC) [1, 2], a substantial number of patients fails to achieve durable remission with modern monoclonal antibodies and small molecules [3]. Regardless of the chosen mode of action, primary non-response is observed in a relevant fraction [4–7]. At the same time, these and numerous studies indicate that the response rates decrease in patients previously exposed to other advanced therapies [8–11]. Since biomarkers predicting individual outcomes of therapy are still lacking [12], this urges the exploration of sequence-dependent efficacy of IBD treatments.

We and others have previously described that real-world treatment trajectories are highly diverse and influenced by factors such as disease activity, patient and physician preferences, but also the order of approval of therapeutics [11, 13]. While the overall decline in treatment efficacy with advancing therapy lines is solidly established, this has most clearly been shown for previous therapy with anti-TNF -antibodies and has usually been explained by an ongoing enrichment of highly refractory cases that progress to further therapy lines.

At the same time, several indications exist that this association is not equally valid for all specific sequences, since the specific modes of action might differentially interfere with sequential pharmacological interventions. For instance, it seems that anti-TNF therapy failure is associated with an enrichment of IL23R^+^ T cells predisposing these patients to subsequent anti-IL23 blockade [14]. Similarly, vedolizumab failure is linked to upregulation of α4β1 integrin [15], which is not directly connected to alternative modes of action. Indeed, some data suggest that first-line therapy with vedolizumab does not impair the success of subsequent anti-TNF therapy as it is the case with anti-TNF therapy [16].

Against this background, this study investigated the sequence-dependent real-world efficacy of anti-TNF antibodies and vedolizumab in a propensity score-weighted single-center cohort of patients with ulcerative colitis. Our data confirm that response rates to second-line vedolizumab after previous anti-TNF therapy are worse than for first-line vedolizumab.

However, no statistically significant difference in clinical or steroid-free remission was observed between anti-TNF therapy used as first-line treatment and as second-line treatment after vedolizumab. While requiring further confirmation, these findings suggest that prior vedolizumab exposure does not appear to significantly compromise the effectiveness of subsequent anti-TNF therapy and may support considering vedolizumab as a first-line treatment option in UC.

## Methods

### Study design and patients

This study was designed as a single-center real-world analysis using propensity score-based inverse probability of treatment weighting (IPTW) to evaluate the efficacy of vedolizumab as first-line therapy versus vedolizumab as second-line therapy after anti-TNFα therapy, and anti-TNFα as first-line therapy versus anti-TNFα as second-line therapy after vedolizumab as first-line therapy. The inclusion criteria for the study were an established diagnosis of ulcerative colitis and ongoing therapy with anti-TNF antibodies (infliximab, adalimumab) or vedolizumab, either as first-line therapy or as second-line therapy after first-line treatment with anti-TNF (for vedolizumab) or vedolizumab (for anti-TNF). Patients with incomplete data, therapies/therapy sequences other than those mentioned or therapies/therapy sequences initiated at other institutions were excluded from the study.

**Fig. 1:**
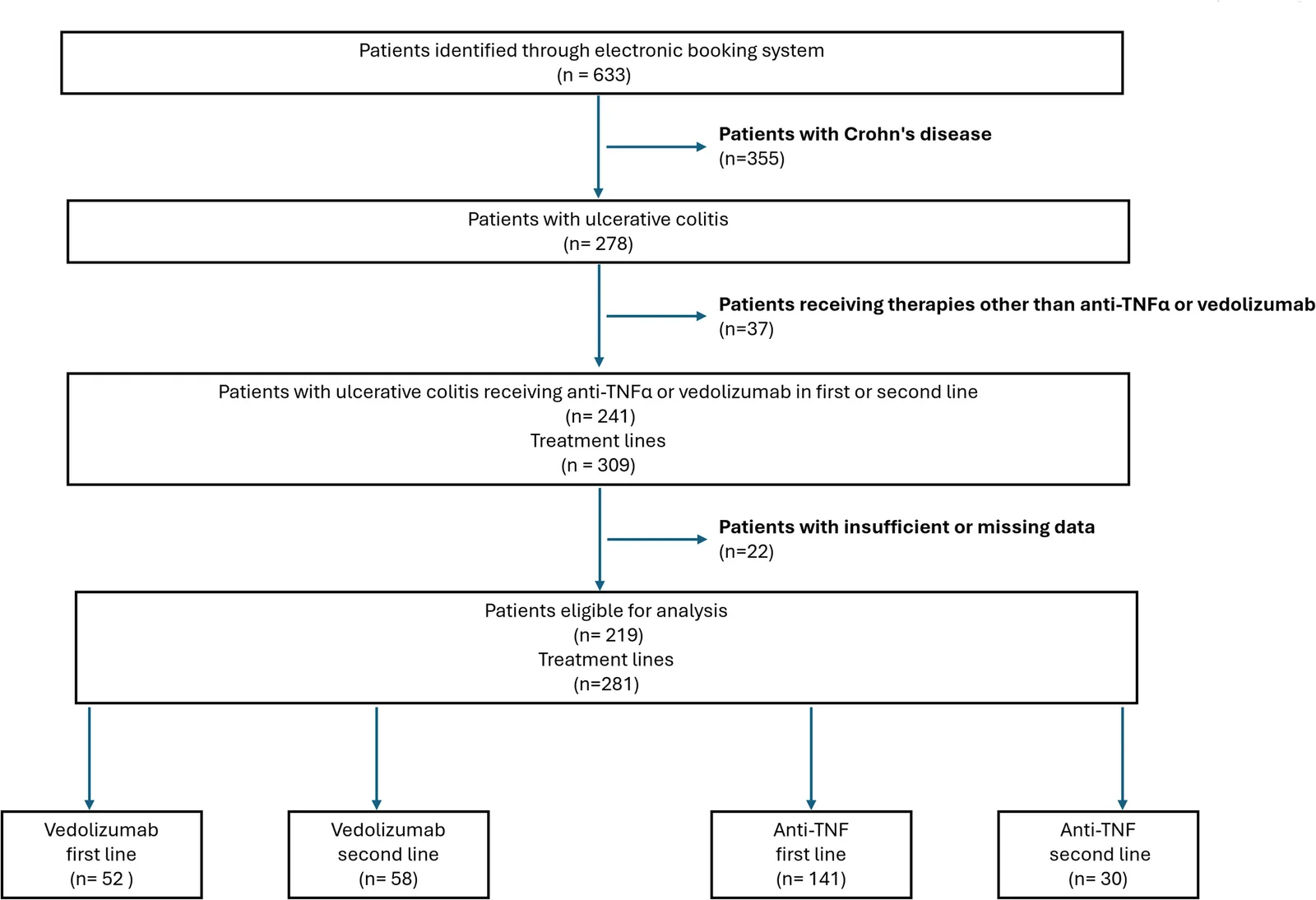
Flow diagram showing the screening process, exclusion and allocation to the different treatment-sequence groups

 A total of 633 patients were initially identified through the electronic booking system (Soarian Scheduling) of the IBD outpatient clinic of the Department of Medicine 1, University Hospital Erlangen. Among these, 278 patients had an established diagnosis of ulcerative colitis. Patients receiving therapies other than anti-TNFα or vedolizumab were excluded (*n* = 37), leaving 241 patients with the predefined treatment options. A further 22 patients were excluded because of insufficient or missing data, resulting in a final cohort of 219 patients eligible for analysis. Patients were included based on initiation of the respective treatment, rather than ongoing treatment at the time of patient identification. The relevant treatment lines were initiated between January 2010 and February 2025 at our study center. A flow chart illustrating the study population is provided in Fig. 1. 

Patients could contribute to more than one treatment-sequence analysis according to their individual treatment history. Thus, the four treatment-sequence cohorts were not mutually exclusive.

### Baseline characteristics and outcomes

The baseline characteristics collected for the patient cohort included age, gender, Montreal classification, partial Mayo score (PMS), concomitant medication and endoscopic Mayo score (EMS). Continuous variables are reported as means with standard error of the mean (SEM), and categorical variables as percentages. Comparisons between groups were performed using the Wilcoxon rank-sum test for continuous variables and the chi-square test for categorical variables.

Clinical remission and steroid-free remission were considered as outcome parameters. Clinical remission was defined as a partial Mayo score (PMS) below 2 and steroid-free remission was defined as clinical remission without corticosteroid use; both were assessed at week 52.

Clinical remission and steroid-free remission at week 52 were assessed using an intention-to-treat approach. Patients who discontinued treatment before week 52 were considered non-responders and were therefore included in the analysis but not considered to have achieved remission. The number of patients included in the intention-to-treat analysis, the number with an observed week-52 outcome, and the number of patients who discontinued treatment before week 52 are reported in Table 1.

**Table 1:** Patient numbers included in the intention-to-treat analysis and week-52 outcome assessment

Treatment group	ITT population	Observed week-52 outcome	Discontinued before week 52
**Anti-TNF first-line**	141	103	38
**Anti-TNF second-line**	30	23	7
**Vedolizumab first-line**	52	37	15
**Vedolizumab second-line**	58	40	18

### Statistical analysis

The aim of the statistical analysis was to compare the treatment sequences described above in terms of clinical and steroid-free remission. The two treatment comparisons were performed independently.

As this study is based on retrospective data, effects of treatment selection bias and confounders are expected. To reduce these, the analysis was performed using inverse probability of treatment weighting (IPTW).

Propensity scores (PS) were estimated using logistic regression, considering the baseline characteristics of age, gender, Montreal classification, and partial Mayo score. These covariates were selected as clinically relevant demographic and disease-related factors that could potentially influence disease severity as well as treatment decision. All covariates were assessed at the initiation of each therapy. For each treatment comparison, the index date was defined as the initiation of the respective therapy in both groups. Patients were weighted based on their propensity scores to create a pseudo-population balanced in terms of baseline characteristics between the treatment groups.

Covariate balance in the pseudo-population was assessed by comparing baseline characteristics before and after weighting. Mean values and standardized mean differences (SMDs) of the original cohort and the weighted pseudo-population are reported. An SMD < 0.1 was considered as an indication of a good balance between the two treatment groups [17].

A weighted logistic regression was performed to analyze the relationship between therapy and the outcome parameters clinical remission and steroid-free remission. The estimation was performed using the svyglm() function from the R package survey, which incorporates robust variance estimators to ensure more accurate standard errors. The previously calculated IPTW weights were included in the model. Results are presented as odds ratios with 95% confidence intervals and p-values. *P* < 0.05 was considered statistically significant.

All analyses were conducted in R (version 4.4.1) using the WeightIt, survey, cobalt, survival, survminer, ggplot2 and dplyr packages.

### Ethics

The study was approved by the Ethics Committee of the Friedrich-Alexander-Universität Erlangen-Nürnberg (20-288-Br).

## Results

### Study population

A total of 219 patients with 281 pre-specified treatment lines were included in the study. 52 patients received vedolizumab as first-line therapy, whereas 58 received vedolizumab as second-line therapy after prior anti-TNF therapy. Anti-TNF therapy was administered as first-line therapy in 141 patients and as second-line therapy in 30 patients after prior vedolizumab therapy.

Among patients receiving second-line anti-TNF therapy, data on the duration of preceding vedolizumab treatment were available for 26 patients. The mean duration of vedolizumab treatment before initiation of anti-TNF therapy was 8.46 ± 13.54 months. Regarding the specific anti-TNF agents, 111 of 141 patients receiving first-line anti-TNF therapy received infliximab and 30 received adalimumab. Among patients receiving second-line anti-TNF therapy after prior vedolizumab, 28 received infliximab and 2 received adalimumab.

Baseline characteristics are summarized in Tables 2 and 3. There were no significant differences in baseline characteristics between patients receiving vedolizumab or anti-TNF in first or second line. However, in addition to some other nominal differences, there was a clear trend towards higher clinical disease activity in patients treated with anti-TNF first-line compared with vedolizumab first-line (Table 4) indicating that therapeutic decisions were probably influenced by clinical presentation and that a direct comparison of these collectives in terms of therapy outcomes might therefore be biased.

**Table 2:** Baseline characteristics of patients treated with vedolizumab

Baseline Characteristics	Vedolizumab first-line	Vedolizumab second-line	p-value
**n**	52	58	
**Age (± SEM)**	49.52 ± 2.43	46.76 ± 2.19	0.405
**Gender**			0.897
**Male**	53.8%	56.9%	
**Female**	46.2%	43.1%	
**Montreal**			0.121
**E1**	19.2%	6.9%	
**E2**	28.8%	39.7%	
**E3**	51.9%	53.4%	
**PMS (± SEM)**	4.15 ± 0.29	4.22 ± 0.28	0.925
**EMS (± SEM)**	1.19 ± 0.14	1.38 ± 0.17	0.566
**Concomitant medication**			
**Corticosteroids**	8.1%	20.0%	0.244
**Immunosuppressants**	0	2.5%	1
**5-ASA**	54.1%	62.5%	0.603

**Table 3:** Baseline characteristics of patients treated with anti-TNF

Baseline Characteristics	Anti-TNF first-line	Anti-TNF second-line	p-value
**n**	141	30	
**Anti-TNF agent**			
**Infliximab**	111 (78.7%)	28 (93.3%)	
**Adalimumab**	30 (21.3%)	2 (6.7%)	
**Age (± SEM)**	44.43 ± 1.24	49.2 ± 3.22	0.174
**Gender**			0.142
**Male**	56.7%	40.0%	
**Female**	43.3%	60.0%	
**Montreal**			0.376
**E1**	11.3%	3.3%	
**E2**	39.0%	46.7%	
**E3**	49.6%	50.0%	
**PMS (± SEM)**	4.82 ± 0.19	4.53 ± 0.49	0.452
**EMS (± SEM)**	1.25 ± 0.09	1.17 ± 0.32	0.704
**Concomitant medication**			
**Corticosteroids**	9.7%	8.7%	1
**Immunosuppressants**	2.9%	4.4%	0.558
**5-ASA**	60.2%	47.8%	0.3938

**Table 4:** Comparison of the baseline characteristics of first-line patients

Baseline Characteristics	Anti-TNF first-line	Vedolizumab first-line	p-value
**n**	141	52	
**Age (± SEM)**	44.43 ± 1.24	49.52 ± 2.43	0.098
**Gender**			0.845
**Male**	56.7%	53.8%	
**Female**	43.3%	46.2%	
**Montreal**			0.238
**E1**	11.3	19.2	
**E2**	39	28.8	
**E3**	49.6	51.9	
**PMS (± SEM)**	4.82 ± 0.19	4.15 ± 0.29	0.056
**EMS (± SEM)**	1.25 ± 0.09	1.19 ± 0.14	0.882
**Concomitant medication**			
**Corticosteroids**	9.7%	8.1%	1
**Immunosuppressants**	2.9%	0%	0.566
**5-ASA**	60.2%	54.1%	0.649

PMS – Partial Mayo Score; EMS – Endoscopic Mayo Score; 5-ASA − 5-aminosalicylic acid; SEM - Standard Error of the Mean; Anti-TNF – Adalimumab or Infliximab

### Covariate balance after IPTW

**Fig. 2:**
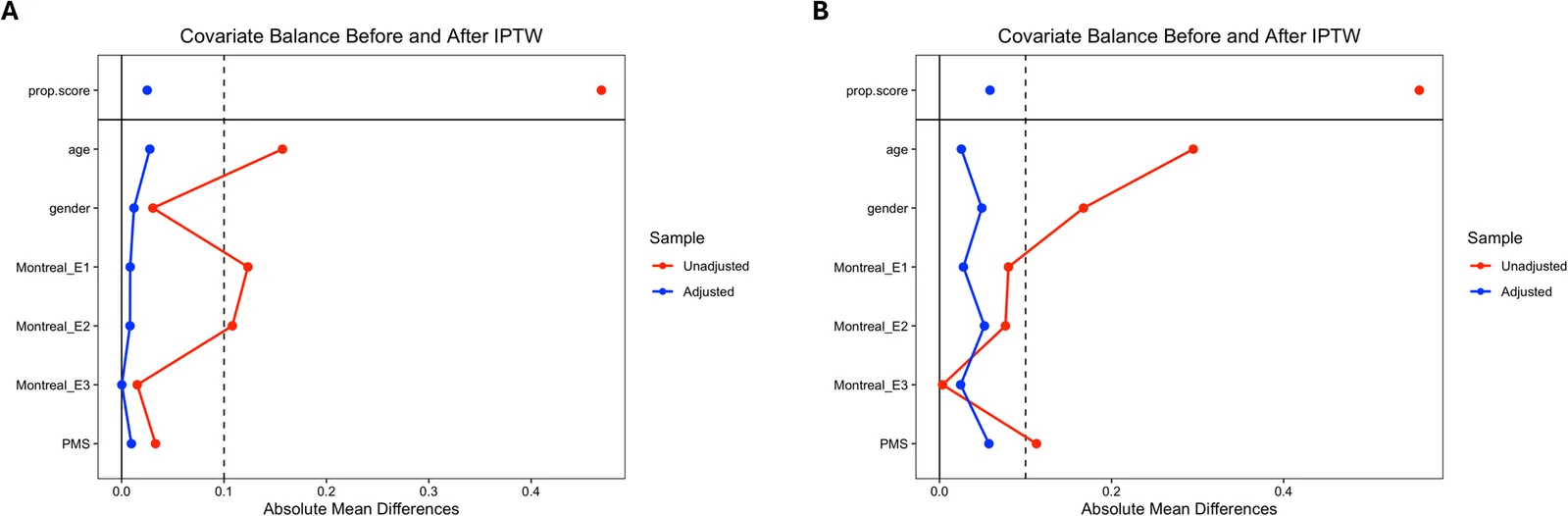
Co-variates in the study population before and after IPTW. Love plots indicating standardized mean differences (SMDs) of co-variates before (red) and after (blue) inverse probability of treatment weighting (IPTW) for comparison of first-line versus second-line Vedolizumab (**A**) and for comparison of first-line versus second-line Anti-TNF-α (**B**) therapy

Thus, we decided to perform IPTW on the cohorts to allow for a more balanced analysis of the therapy outcomes. Before applying IPTW, relevant differences were observed regarding age, PMS, gender and Montreal classification. After weighting, covariate balance improved substantially, with all standardized mean differences below 0.1, indicating adequate balance across groups. Details are presented in Supplementary Table 1. Figure 2 provides a graphical representation of the change in SMD before and after IPTW.

### Clinical remission

**Fig. 3:**
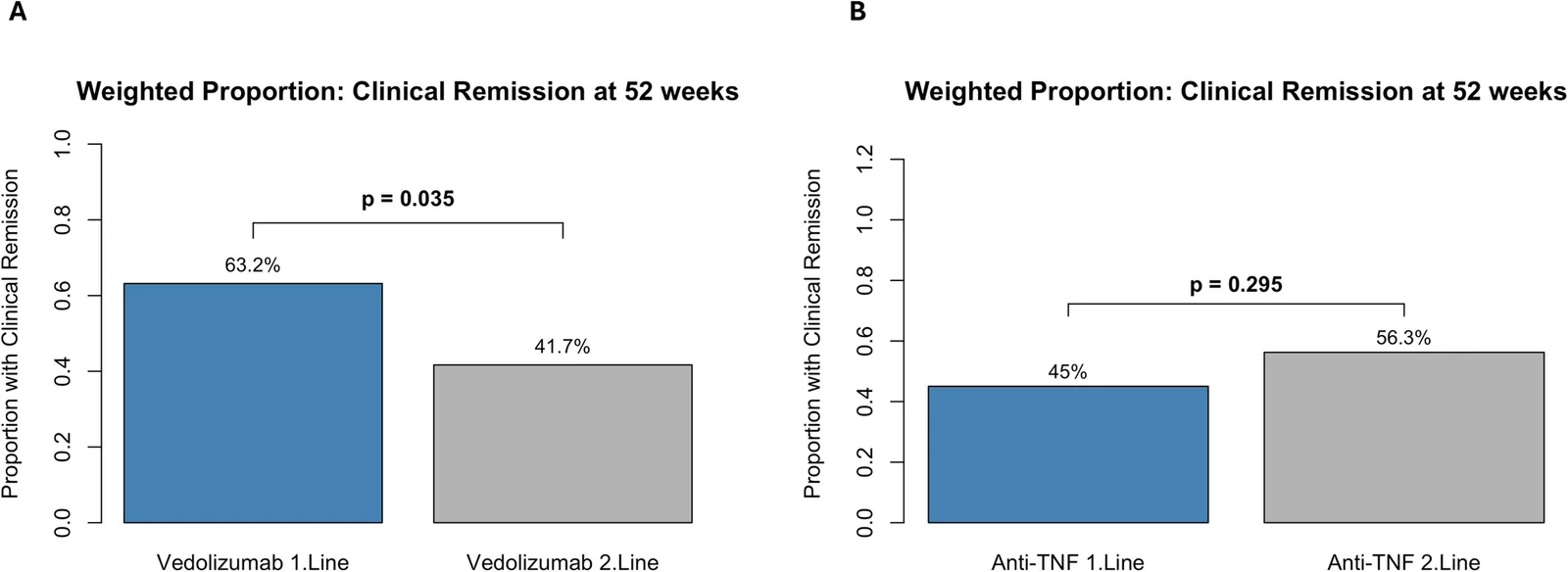
Clinical remission at 52 weeks. Bar chart showing the proportion of patients achieving clinical remission at 52 weeks in the IPTW-weighted cohort, comparing first- and second-line Vedolizumab therapy (**A**) and comparing first- and second-line Anti-TNF-α therapy (**B**)

Within the weighted pseudo-population, we examined the extent to which clinical remission at 52 weeks was achieved within the different groups. Consistent with previous literature, clinical remission at week 52 was significantly higher in patients receiving vedolizumab as first-line therapy than in those receiving second-line therapy (OR 2.40, 95% CI 1.08–5.36; *p* = 0.035). Patients receiving vedolizumab as first-line therapy achieved clinical remission in 63.2%, whereas patients receiving vedolizumab as second-line therapy achieved clinical remission in 41.7% (Fig. 3A). However, no significant difference was observed between first-line and second-line anti-TNF therapy (45.0% vs. 56.3%; OR 0.64, 95% CI 0.28–1.48; *p* = 0.295; Fig. 3B).

### Steroid-free remission

**Fig. 4:**
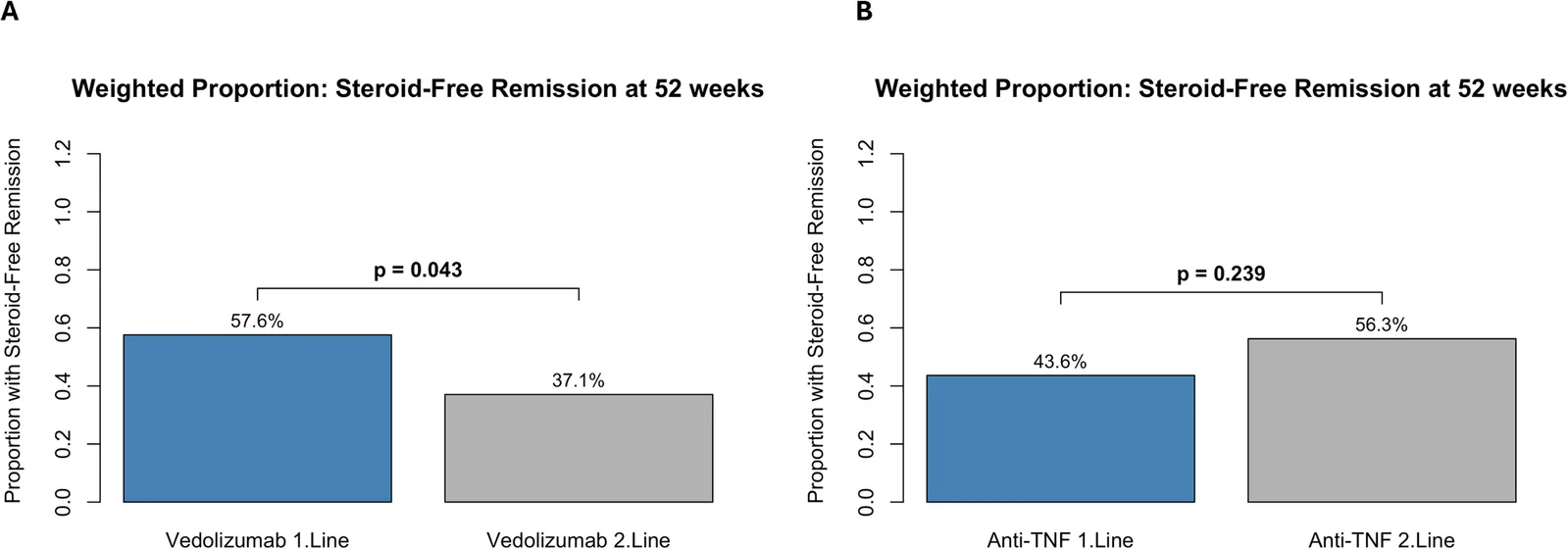
Steroid-free remission at 52 weeks. Bar chart showing the proportion of patients achieving steroid-free remission at 52 weeks in the IPTW-weighted cohort, comparing first- and second-line Vedolizumab therapy (**A**) and comparing first- and second-line Anti-TNF-α therapy (**B**)

Similarly, steroid-free remission was analyzed in the pseudo-population. Steroid-free remission was significantly higher with vedolizumab as first-line therapy than with second-line therapy (57.6% vs. 37.0%; OR 2.31, 95% CI 1.04–5.14; *p* = 0.043; Fig. 4A).

Among anti-TNF-treated patients, no significant difference was observed between first-line and second-line treatment (43.6% vs. 56.3%; OR 0.60, 95% CI 0.26–1.40; *p* = 0.239; Fig. 4B).

### Infliximab sensitivity analysis

In a sensitivity analysis restricted to patients receiving infliximab, clinical remission at week 52 was achieved in 48.5% of patients receiving first-line infliximab compared with 57.7% receiving second-line infliximab after prior vedolizumab exposure (OR 0.69; 95% CI 0.29–1.65; *p* = 0.405). Similarly, in the infliximab-restricted sensitivity analysis, steroid-free remission at week 52 was achieved in 46.7% of patients receiving first-line infliximab compared with 57.7% receiving second-line infliximab after prior vedolizumab exposure (OR 0.64; 95% CI 0.27–1.53; *p* = 0.318). These findings were consistent with the results of the overall anti-TNF analysis. The infliximab-restricted sensitivity analysis was also adjusted using IPTW.

### Persistence on therapy

**Fig. 5:**
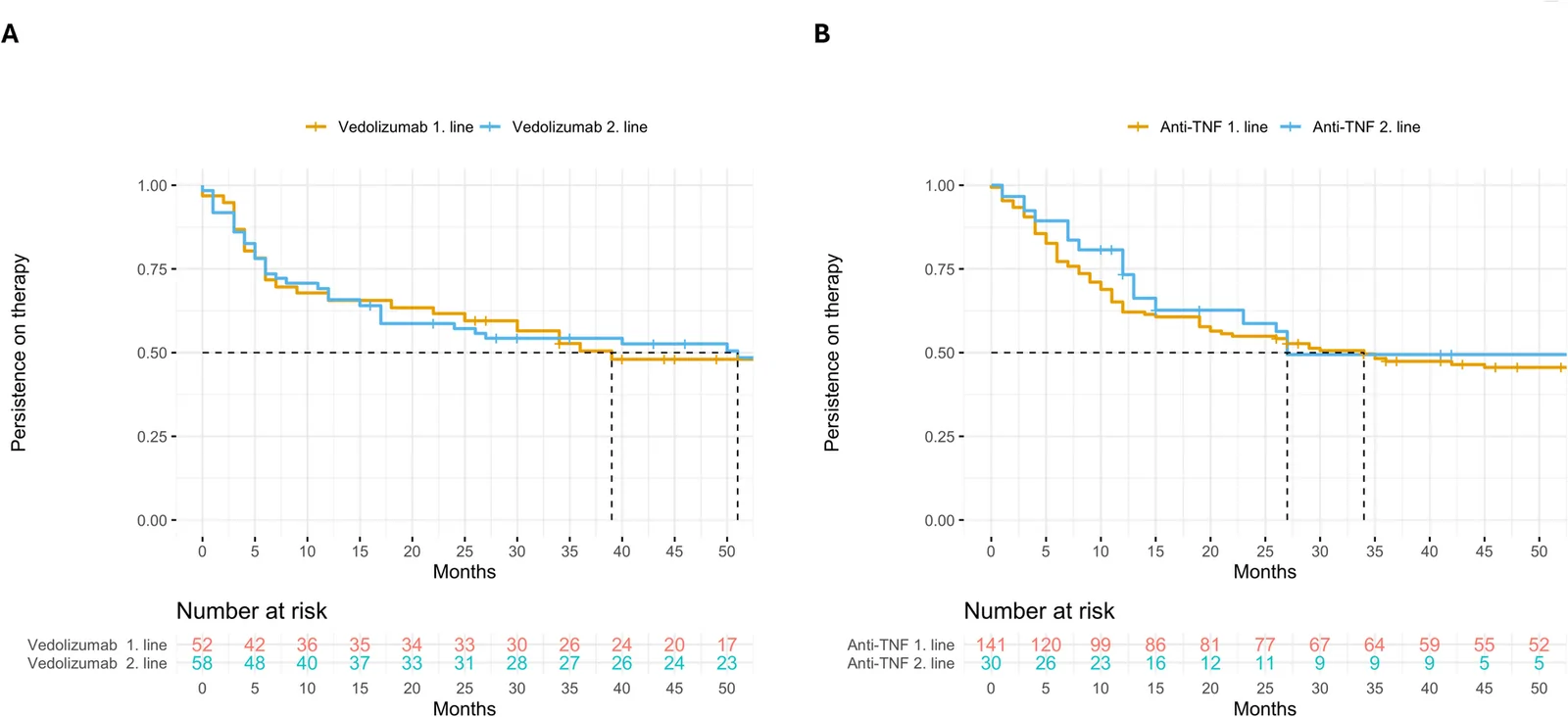
Persistence on therapy. Kaplan-Meier plots showing persistence on therapy in weighted population on Vedolizumab first-line vs. Vedolizumab second-line (**A**) and Anti-TNF-α first-line vs. second-line (**B**). Additionally, the numbers at risk for the unweighted study population are shown below the Kaplan–Meier curves

To examine treatment persistence between the groups, Kaplan-Meier curves were plotted (Fig. 5). Kaplan-Meier analyses showed no significant difference in treatment persistence between vedolizumab as first-line and second-line therapy (log-rank *p* = 0.45). Similarly, no significant difference was observed between first-line and second-line anti-TNF therapy (log-rank *p* = 0.74).

## Discussion

There is currently no broad consensus on the sequential prioritization of medical therapies for the treatment of ulcerative colitis. Phase III trials are usually performed vs. placebo and only few head-to-head-trials exist [18, 19]. Thus, while some guidelines have recently started to indicate certain degrees of preference in specific conditions [20], others do still list all available compounds in alphabetical order to reflect this lack of data [21, 22]. Consequently, treatment decisions are frequently based on individual preferences or experience.

Given the well-established observation that patients with previous exposure to advanced therapies respond worse to subsequent lines of treatment compared with bio-naïve patients [4–7, 23], two apparent strategies emerge to improve outcomes of IBD therapy along the therapeutic journey: First, individual treatment algorithms might be optimized by specifically predicting the most promising personalized therapeutic approach based on biomarkers [24]. However, while various strategies in this regard have been proposed in the past [25–27], none of them has so far entered clinical practice [28, 29]. This quest is therefore still ongoing and it is currently unclear whether it will ever be successful. Second, treatment algorithms with superior overall outcomes might be identified on population level to generate evidence for treating patients along a rationally designed pathway.

Our current study was designed to contribute to this approach. It follows up on previous data from the EVOLVE trial, a retrospective medical chart review, including a total of 1095 patients with CD or UC [16]. While several randomized trials and real-world cohorts have shown that the efficacy of vedolizumab is clearly reduced after previous anti-TNF exposure [4, 30–32], a sub-analysis of this study showed that therapy persistence, clinical response and clinical remission to anti-TNF-antibody therapy for up to six months in UC and CD was similar in first-line compared to second-line after previous vedolizumab therapy. Our current results confirm both observations in a single-center real-world cohort: Decreased efficacy of vedolizumab following previous anti-TNF and no statistically significant reduction in anti-TNF effectiveness following prior vedolizumab exposure. They extend the data from the EVOLVE trial by addressing clinical remission after 12 months and by adding steroid-free remission as a further outcome. Our observations indicate that no statistically significant reduction in the effectiveness of subsequent anti-TNF therapy was detected following prior vedolizumab exposure. Although we report on an overall cohort of 219 patients, the subgroup of patients receiving anti-TNF after previous vedolizumab consisted of only 30 patients. However, this is still clearly more than previously reported (12 patients after six months). It should be noted that another retrospective trial found superior treatment persistence in second-line vedolizumab after anti-TNF compared with second-line anti-TNF after vedolizumab [33]. However, no​ comparison of first- vs. second-line efficacy of these agents was performed in this case.

Several limitations have to be considered, when interpreting our data: First, they come from a single center and are retrospective in nature and therefore require confirmation in prospective multi-center studies. Second, related to the retrospective design, we were not able to include more objective endpoints such as endoscopic or biochemical remission. Although our study provides insights into clinical outcomes, we could not objectively assess endoscopic or biochemical disease control. Consequently, further studies including objective measures of disease activity would be useful to confirm our findings. Third, given the only small number of patients with CD treated with second-line vedolizumab after first-line anti-TNF at our center in the study period, we had to confine our analyses to UC. These can therefore not be generalized to overall IBD. Fourth, the anti-TNF cohort comprised both infliximab and adalimumab, with only two patients receiving adalimumab in the second-line group. Therefore, a meaningful drug-specific analysis for adalimumab was not possible. However, an infliximab-restricted sensitivity analysis yielded results consistent with the primary anti-TNF analysis. Fifth, although we used IPTW, residual confounding cannot be completely excluded. Our IPTW analysis included age, sex, Montreal classification, and partial Mayo score as covariates; however, other clinically relevant factors may also influence disease severity and treatment decisions. Moreover, differences in prior treatment history between the first- and second-line anti-TNF groups may have contributed to the numerically higher remission rates observed in the second-line group. And sixth, it has to be acknowledged that the design including different sequences of two modes of action (anti-TNF, anti-integrin) does only insufficiently capture the current reality of IBD treatment, since various other sequences, in particular involving anti-(IL12/)23 antibodies, Jak inhibitors and S1PR1 modulators, are conceivable and would need to be included into a fully comprehensive study on the question of ideal therapeutic algorithms. Yet, while these aspects provide important cues for guiding future research efforts, we feel that our data contribute important insights into rational sequential UC therapy.

From a clinical standpoint, these findings should also be seen in the context of the VARSITY head-to-head-trial of vedolizumab vs. adalimumab [19] and various real-world reports that overall suggest better long-term outcomes in the treatment of UC with first-line vedolizumab compared with first-line anti-TNF [16, 34, 35]. Thus, collectively, except for patients with highly severe disease activity, where the probability of response to vedolizumab has been reported to decrease [36], we conclude that both the efficacy in first-line, the excellent safety profile [37] as well as the absence of a statistically significant reduction in subsequent anti-TNF effectiveness following prior vedolizumab exposure, are factors that are compatible with considering vedolizumab as a first-line treatment option of UC. However, as our study did not directly compare vedolizumab-first with anti-TNF-first-treatment strategies, these findings do not establish a preferred treatment sequence. Further research is needed to further evaluate these findings and to determine the optimal treatment sequence.

## Conclusions

While requiring further confirmation in prospective trials, our data demonstrate that vedolizumab is more effective when used as first-line therapy than as second-line therapy following prior anti-TNF exposure, while no statistically significant reduction in the effectiveness of subsequent anti-TNF therapy was detected following prior vedolizumab exposure. These findings suggest that prior vedolizumab exposure does not appear to significantly compromise the effectiveness of subsequent anti-TNF therapy. Overall, these results are compatible with considering vedolizumab as a first-line treatment option in UC. However, trials directly comparing vedolizumab-first and anti-TNF-first treatment strategies are needed to determine the optimal treatment sequence.

## Supplementary information

Below is the link to the electronic supplementary material.

Supplementary Material 1

## Data Availability

All data underlying this study are available from the authors upon reasonable request.

## Abbreviations

CD: Crohn’s disease
EMS: endoscopic Mayo score
IBD: inflammatory bowel disease
IPTW: inverse probability of treatment weighting
PMS: partial Mayo score
PS: propensity score
SEM: standard error of the mean
SMD: standardized mean differences
TNF: tumor necrosis factor
UC: ulcerative colitis
