# Supplementary material for: Sequence-dependent real-world efficacy of anti-TNF antibodies and vedolizumab in ulcerative colitis: a propensity score-weighted cohort study
Source: BMC Gastroenterol. 2026 Nov 14;26:760. doi: 10.1186/s12876-026-05560-4 (PMC13769175; doi:10.1186/s12876-026-05560-4)
Supplement: Supplementary file 1 [file 12876_2026_5560_MOESM1_ESM.docx]

**Supplementary Table 1: Comparison of Patient Characteristics before and after IPTW**

|  | **Original Cohort (pre-IPTW)** | | | **Pseudo Cohort (post-IPTW)** | | |
| --- | --- | --- | --- | --- | --- | --- |
| **Characteristics** | **Vedolizumab  first-line** | **Vedolizumab second-line** | **SMD** | **Vedolizumab  first-line** | **Vedolizumab second-line** | **SMD** |
| **Age** | 49.52 | 46.76 | 0.161 | 48.36 | 48.84 | -0.028 |
| **PMS** | 4.15 | 4.22 | -0.033 | 4.25 | 4.23 | 0.01 |
| **Gender** |  |  | 0.031 |  |  | 0.012 |
| **Male** | 53.8% | 56.9% |  | 56.2% | 57.4% |  |
| **Female** | 46.2% | 43.1% |  | 43.8% | 42.6% |  |
| **Montreal** |  |  | 0.123 |  |  | 0.009 |
| **E1** | 19.2% | 6.9% |  | 12.9% | 13.8% |  |
| **E2** | 28.8% | 39.7% |  | 35.3% | 34.4% |  |
| **E3** | 51.9% | 53.4% |  | 51.8% | 51.8% |  |

|  | **Original Cohort (pre-IPTW)** | | | **Pseudo Cohort (post-IPTW)** | | |
| --- | --- | --- | --- | --- | --- | --- |
| **Characteristics** | **Anti-TNF first-line** | **Anti-TNF second-line** | **SMD** | **Anti-TNF first-line** | **Anti-TNF second-line** | **SMD** |
| **Age** | 44.43 | 49.2 | -0.295 | 45.29 | 45.7 | -0.025 |
| **PMS** | 4.82 | 4.53 | 0.113 | 4.78 | 4.92 | -0.057 |
| **Gender** |  |  | 0.167 |  |  | -0.049 |
| **Male** | 56.7% | 40% |  | 53.7% | 48.8% |  |
| **Female** | 43.3% | 60% |  | 46.3% | 51.2% |  |
| **Montreal** |  |  | 0.08 |  |  | 0.052 |
| **E1** | 11.3% | 3.3% |  | 9.9% | 7.2% |  |
| **E2** | 39% | 46.7% |  | 40.6% | 45.8% |  |
| **E3** | 49.6% | 50% |  | 49.5% | 47% |  |

IPTW - **Inverse Probability of Treatment Weighting**; SMD - **Standardized Mean Difference; PMS – Partial Mayo Score; Anti-TNF – Adalimumab or Infliximab**
